# Left Atrial Appendage Occlusion: What Are the Options and Where is the Evidence?

**DOI:** 10.19102/icrm.2018.090402

**Published:** 2018-04-15

**Authors:** Raghuram Chava, Mohit K. Turagam, Dhanunjaya (DJ) Lakkireddy

**Affiliations:** ^1^Department of Internal Medicine, MedStar Harbor Hospital, Baltimore, MD, USA; ^2^Section of Electrophysiology, Mount Sinai Hospital, New York, NY, USA; ^3^Division of Cardiovascular Diseases, Cardiovascular Research Institute, University of Kansas Hospital and Medical Center, Kansas City, KS, USA

**Keywords:** Amulet, anticoagulation, left atrial appendage, thromboembolism, WATCHMAN™

## Abstract

Left atrial appendage occlusion (LAAO) has emerged as an effective site-directed therapy in patients with nonvalvular atrial fibrillation (AF) for stroke prevention, who are ineligible for long-term oral anticoagulation. The objective of this study was to assess the safety, efficacy, and availability of LAAO devices by reviewing the literature and to review the development and effectiveness of LAAO by the transcatheter approach with plugging devices such as WATCHMAN™ (Boston Scientific, Natick, MA, USA); AMPLATZER™ Cardiac Plug and AMPLATZER™ Amulet™ (Abbott Laboratories, Chicago, IL, USA); and the LARIAT^®^ Suture Delivery Device (SentreHEART, Redwood City, CA, USA), which features an entirely unique hybrid (endocardial and epicardial) approach in closing the left atrial appendage (LAA). The conducted literature review ultimately revealed a substantial body of literature supporting the safety and efficacy of various LAAO strategies, including endocardial, epicardial, and hybrid approaches, in AF patients who are not eligible for long-term oral anticoagulant use. Specifically, the most attractive population suitable for LAA closure appears to be patients at high risk for ischemic stroke with a longer life expectancy but a moderate-to-high bleeding risk with long-term oral anticoagulation. The benefit of LAA closure in reducing the incidence of stroke in patients with nonvalvular AF has been evolving gradually, and we are confident that this new field of percutaneous LAA closure will continue to emerge as a game-changer in the treatment of AF.

## Introduction

Atrial fibrillation (AF) is the most common persistent arrhythmia, affecting between six and seven million people in the United States alone and projected to affect between 12 and 14 million people by 2050.^1^ It is associated with a four- to fivefold increased risk of ischemic stroke with significant morbidity and mortality.^2^ It has been suggested by the American College of Cardiology and the American Heart Association that systemic anticoagulation with vitamin K antagonists (VKAs) such as warfarin or novel non-VKA oral anticoagulants (NOACs) be used for the prevention of ischemic stroke and systemic thromboembolism in appropriate AF patients with CHA_2_DS_2_-VASc scores ≥ 2 [level of evidence (LOE) A]^3^; however, despite this recommendation, these medications remain underutilized.^4–7^

Notably, NOACs do not require ongoing monitoring and have proven to be noninferior or superior to warfarin in preventing systemic embolism and ischemic stroke. Despite these benefits, however, major practical challenges to systemic oral anticoagulation (OAC) with NOACs include compliance, major bleeding, side effects, drug–drug and drug–diet interactions, and a lack of antidotes for NOACs other than dabigatran [idarucizumab (Praxbind^®^; Boehringer Ingelheim, Ingelheim am Rhein, Germany) is available for use during emergency situations to reverse the anticoagulation effects of dabigatran].

Furthermore, there are substantial data demonstrating that 47% of patients stop OAC within two years of commencement, with discontinuation more frequently observed in patients with a prior history of bleeding.^8^ The significant level of practical difficulty associated with the continuation of anticoagulation in AF patients at a high risk of bleeding has forced clinicians to search for alternative options to prevent ischemic stroke in this patient group.

It is known that the left atrial appendage (LAA) is the predominant site (in > 90% of cases) of thrombus formation in AF patients with ischemic stroke or those who have been diagnosed with thrombus or emboli.^9^ AF leads to a loss of contractile function of the LAA by causing mechanical dysfunction of the atrial tissue, which leads to local stasis and thrombus formation that may be the source of the emboli that result in cases of ischemic stroke. Mechanical LAA closure is a current promising alternative for reducing systemic thromboembolic events in these patients without increasing the risk of bleeding.

## Available methods for left atrial appendage occlusion

Currently, several devices are available that are geared towards the LAA for the management of stroke and systemic thromboembolism. These devices are classified on the basis of the accompanying technique and the nature of their use as either endocardial, epicardial, or a combination of both. Endocardial transcatheter closure is the most widely used and tested method for the closure of LAAs. The WATCHMAN™ (Boston Scientific, Natick, MA, USA) and the AMPLATZER™ Amulet™ (Abbott Laboratories, Chicago, IL, USA)—the latter of which gained CE mark approval in January 2013 but which is still part of a clinical trial in the United States—are two devices proven to be effective in this approach.^10–13^ A third device, the percutaneous LAA transcatheter occlusion (PLAATO) system (Appriva Medical Inc., Sunnyvale, CA, USA), was previously tested but was discontinued in 2006.^14^ In addition to these options, implantation of the LARIAT^®^ device (SentreHeart, Inc., Redwood City, CA, USA) is a procedure that involves a combined endo–epicardial ligation of the LAA through a surgical suture. This procedure has also demonstrated good results.^15^ Epicardial approaches involve the placement of an epicardial clip, such as the ATRICLIP^®^ device (AtriCure, Mason, OH, USA), at the base of the LAA in patients. A completed clinical trial has demonstrated safety and efficacy of the ATRICLIP^®^ device (AtriCure, Mason, OH, USA) for LAA occlusion (LAAO) in patients undergoing concomitant cardiac surgery, with greater than 95% success and durability in the short-term, as determined by imaging.^16^

## Transcatheter closure strategies

### Percutaneous left atrial appendage transcatheter occlusion

The PLAATO system (Appriva Medical Inc., Sunnyvale, CA, USA) was the first transcatheter device developed for LAAO. The device, introduced in 1998, consisted of an expanded polytetrafluoroethylene membrane covering a self-expanding nitinol cage. The European PLAATO trial^17^ was the first experimental multicenter human study involving this device and included 64 patients who were not candidates for warfarin but who had a high risk of thromboembolism.^18^ The trial results indicated the anatomic closure and safety of the device were excellent (residual flow ≤ 3 mm in 98% of patients). Additionally, the observed rate of stroke or transient ischemic attack was 3.8% per year in comparison with an expected rate of 6.6% per year based on the CHADS_2_ scores of the study population at the five-year follow-up mark. This study was halted prematurely during the follow-up phase, however, because of some financial considerations.^14^ Though other, smaller studies have also attempted to quantify the device’s role in terms of stroke management,^14,19,20^ it was ultimately withdrawn from the marketplace in 2006.

### WATCHMAN™

The WATCHMAN™ (Boston Scientific, Natick, MA, USA) is the first percutaneous LAA occlusive device approved by the US Food and Drug Administration (FDA) for the prevention of thromboembolism by addressing the LAA in patients with nonvalvular AF.

**Figure 1:**
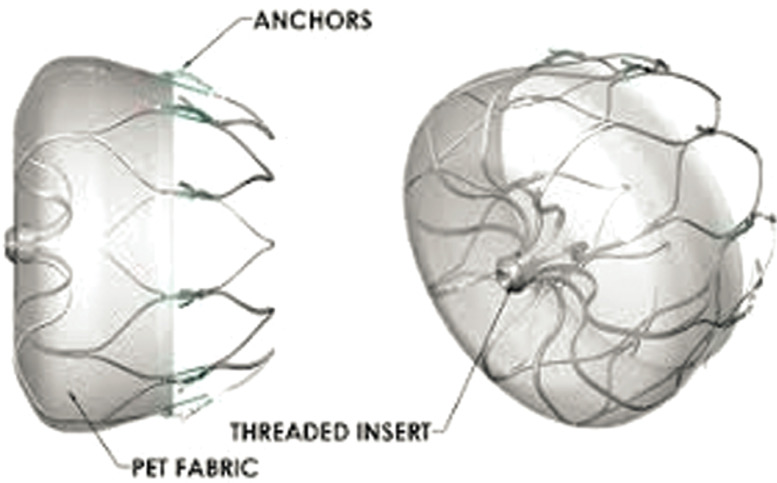
The WATCHMAN™ device (Boston Scientific, Natick, MA, USA) this device is made of a self-expanding nitinol frame with a polyethylene terephthalate fabric cap. Distal tines secure the device within the LAA trabeculae. It is fully retrievable prior to release from the delivery cable. The device’s length is approximately equal to its diameter. Device size is selected on the basis of the largest diameter of the LAA ostium, which is measured by drawing a line from the mitral valve annulus across to the ridge of the left upper pulmonary vein, perpendicular to the planned axis of the delivery sheath. Alternatively, the LAA ostium can be measured from the mitral valve annulus to a point ≈ 2 cm distal from the tip of the left upper pulmonary vein ridge. Image courtesy of Boston Scientific.

***Device characteristics***. The WATCHMAN™ (Boston Scientific, Natick, MA, USA) consists of a self-expanding nitinol frame and a membrane cover **(Figure 1)**. It is delivered through a percutaneous approach via a 14-French sheath placed in the LAA, guided by fluoroscopy and transesophageal echocardiography (TEE).

***Clinical data and efficacy***. The WATCHMAN™ device (Boston Scientific, Natick, MA, USA) went through two randomized control trials to prove its safety and efficacy as part of a prolonged premarket pathway. Both the WATCHMAN™ LAA System for Embolic Protection in Patients With AF (PROTECT AF) and Evaluation of the WATCHMAN™ LAA Closure Device in Patients with AF Versus Long-term Warfarin Therapy (PREVAIL) trials are noninferiority trials that compared the use of the WATCHMAN™ device (Boston Scientific, Natick, MA, USA) versus warfarin in patients with AF who were candidates for long-term anticoagulation. The patients of each study were randomly assigned in a 2:1 fashion^21^ to either a device implantation group or a warfarin-only therapy (control) group. The device implantation group was treated with warfarin and aspirin for six weeks following implantation and was scheduled to undergo a follow-up TEE scan. At that time, if no thrombus or peridevice leak < 5 mm was observed using transthoracic echocardiography (TTE), then the warfarin therapy was discontinued and the aspirin and clopidogrel were prescribed for an additional five months, followed by aspirin indefinitely; otherwise, they were continued on warfarin until another follow-up TEE scan was performed that yielded more favorable results.

***Results and efficacy of the PROTECT AF trial***. The WATCHMAN™ device (Boston Scientific, Natick, MA, USA) was noninferior to warfarin in the PROTECT AF trial^12^ for the coprimary endpoints of cardiovascular/unexplained death, any stroke, or systemic embolism at 1,065, 1,588, and 2,621 patient-years of follow-up, respectively, and it met the criterion for superiority. Furthermore, at a late follow-up point of 3.8 years (2,621 patient-years), all-cause mortality was significantly reduced [hazard ratio (HR): 0.66; 95% confidence interval (CI): 0.45–0.98; p = 0.04] **(Table 1)**.^22^ There was a significant improvement in quality of life and decreased mortality also noted due to a decreased bleeding rate in the group of patients treated with WATCHMAN™ (Boston Scientific, Natick, MA, USA) in comparison with the group assigned to receive warfarin therapy. In PROTECT AF, landmark analyses confined to the periods after the procedure and after the termination of warfarin and clopidogrel therapy in the device arm demonstrated that the primary endpoint of ischemic stroke rates was similar in both the warfarin and device arms, respectively.^10,11^ Analysis from this study supports the hypothesis that LAAO reduces the risk of ischemic stroke in the absence of OAC.

The main limitations of the PROTECT AF trial include: (1) the inclusion of patients with a CHADS_2_ score of ≥ 1, which leads to a higher rate of hemorrhagic stroke in the warfarin-treated patients in comparison with in previous experiences; and (2) a higher rate of the major safety endpoint at 18 months (excessive bleeding or a procedure-related complication) in the patients who received the WATCHMAN™ device (Boston Scientific, Natick, MA, USA) versus those in the warfarin group [risk ratio (RR): 1.69; 95% credible interval: 1.01–3.19], aided by procedure-related ischemic stroke and pericardial effusions occurring in the first seven days after the procedure.

***Results and efficacy of the PREVAIL trial***. In this study, at 18 months of follow-up, the coprimary endpoint data of cardiovascular death, any stroke, or systemic embolism were numerically similar between the warfarin and WATCHMAN™ groups, but the use of the device in the latter led to the nonachievement of the prespecified noninferiority margin of 1.75, because of the higher value of the 95% CI for the 18-month-rate ratio **(Table 1)**. However, device implantation was found to be noninferior to warfarin use^23,24^ if the fact of the coprimary endpoint (ischemic stroke or systolic embolism) occurring after seven days after the procedure was considered.

In the PREVAIL trial, the implantation of WATCHMAN™ (Boston Scientific, Natick, MA, USA) by new operators was not associated with an increased risk or decreased rates of implantation success or an increased rate of major adverse events. Continuing-access protocol (CAP) registry results that followed the conclusion of the PROTECT AF trial are consistent with the improved safety profile indicated in the PREVAIL trial.^12,25^ Additional longer-term follow-up data presented in consideration of the PROTECT AF study further indicated a mortality benefit with WATCHMAN™ (Boston Scientific, Natick, MA, USA) use.^22^

***ASA Plavix Feasibility Study with WATCHMAN™ LAA Closure Technology***. The ASA Plavix Feasibility Study with WATCHMAN™ LAA Closure Technology (ASAP) trial was a multicenter prospective nonrandomized study of LAA closure completed with the WATCHMAN™ device (Boston Scientific, Natick, MA, USA) in patients with nonvalvular AF and a CHADS_2_ score of > 1 who were ineligible for even short-term OAC but who were eligible for six months’ treatment with a thienopyridine antiplatelet agent (ie, clopidogrel or ticlopidine) and lifelong aspirin.^26^ Patients with a history of prior bleeding were clinically and closely observed using the WATCHMAN™ device (Boston Scientific, Natick, MA, USA). Importantly, stroke prevention in patients with contraindications for anticoagulation has been a challenging task for clinicians; this study enrolled 150 patients who were followed up for 14.4 months ± 8.6 months, with findings indicating the risk of stroke or systemic embolism to be significantly reduced to 2.3% rather than 7.3% (the level expected based on CHADS_2_). ASAP represents the first prospective study of LAA closure completed with the WATCHMAN™ device (Boston Scientific, Natick, MA, USA) in patients with contraindications for even short-term anticoagulation. The main limitations of this study were its small study cohort, expected stroke rate, and lack of randomization. Additional five-year follow-up data were also reported in a separate study.^27^

In addition, a systemic review and meta-analysis of seven randomized controlled trials (n = 73,978) for the prevention of stroke by LAAO devices and NOACs in comparison with warfarin in patients with nonvalvular AF concluded that the device is a reasonable noninferior alternative to warfarin for stroke prevention, with similar efficacy endpoints but more complications (OR: 0.79; 95% CI: 0.65–0.97; p = 0.01).^28^ Bleeding and device- or procedure-related complications were the major safety endpoints of the studies considered. The proportion of safety endpoints was 5% in the NOAC group versus 10.7% in the device group. This analysis favored NOAC use over with warfarin use (OR: 0.79; CI: 0.65–0.97; p = 0.026), but indicated that device use was associated with more complications as compared with warfarin use (OR: 1.85; 95% CI: 1.14–3.01; p = 0.012). It was advised that the WATCHMAN™ device (Boston Scientific, Natick, MA, USA) be used cautiously due to the safety concerns raised.^28^ Furthermore, it was suggested that a larger adequate dataset is required to define the appropriate postprocedural medical regimen following LAAO device implantation in patients with AF who have contraindications to OAC.

***EWOLUTION registry***. The EWOLUTION study/registry was designed as a prospective, multicenter, non-randomized cohort study that included a total of 1,025 patients who were scheduled to receive a WATCHMAN™ device (Boston Scientific, Natick, MA, USA) in a total of 47 centers in 13 countries; ultimately, however, only 1,020 patients were included (five were excluded). Eligible patients were enrolled consecutively to represent real-life practice and to avoid any selection bias. The device was successfully deployed in 1,004 of the 1,020 patients (98.5%); 15 patients demonstrated unfavorable anatomy for device implantation, and one patient’s implant status was unknown. The major reasons for exclusion (five patients) and implant failure (15 patients, 1.5%) were unfavorable anatomy or a mismatch between the size of the device and the LAA, though, as previously stated, implant status was unknown in one patient. Procedural closure was successfully achieved in 99.3% of the implanted patients (defined as no or < 5 mm of residual flow assessed via periprocedural TEE).^29^ Eighty-four serious adverse events (SAEs) occurred in 73 patients within 30 days of the implant procedure, with a mortality rate of 0.7%. Thirty-four SAEs in 32 of the 73 patients were device- or procedure-related, while the remaining 50 SAEs in 48 of the 73 patients were unrelated to the procedure or device. This study therefore demonstrated that LAA closure can be successfully and relatively safely performed in patients deemed unsuitable for OAC.^29^

### AMPLATZER™ Cardiac Plug

**Figure 2:**
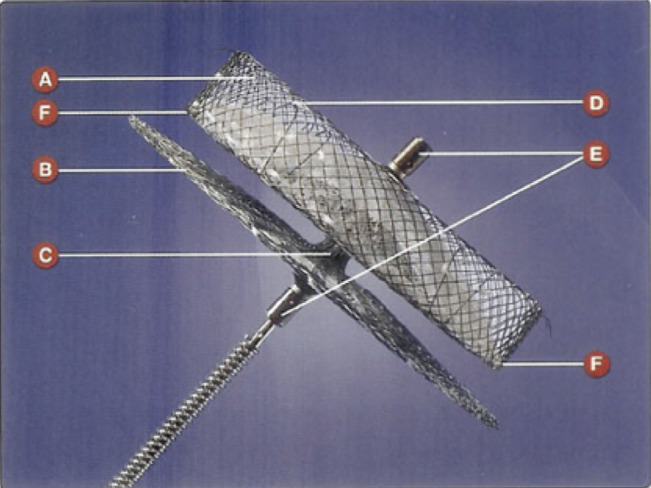
The AMPLATZER™ Cardiac Plug (Abbott Laboratories, Chicago, IL, USA). This device is a self-expanding nitinol mesh that consists of a distal lobe and proximal disk, connected by a short central waist and covered with a sewn polyester patch. During use, the distal lobe hooks around its circumference and anchors the device within the appendage, with the disk positioned proximally and occluding the mouth of the LAA. A: lobe; B: disc; C: waist; D: stabilizing wire; E: radiopaque markers; F: radiopaque threads.

The AMPLATZER™ Cardiac Plug (Abbott Laboratories, Chicago, IL, USA) consists of a self-expanding nitinol mesh comprising a distal lobe and proximal disk connected by a short central waist and covered with a sewn polyester patch **(Figure 2)**.^30,31^ The device is delivered from the femoral vein via transseptal puncture guided by fluoroscopy and TEE. The proximal disk of the AMPLATZER™ Cardiac Plug (Abbott Laboratories, Chicago, IL, USA) covers the mouth of the LAA from within the left atrium, differing from the WATCHMAN™ device (Boston Scientific, Natick, MA, USA), which occludes from within the appendage itself. It is currently in use in Europe, but has not yet received approval in the US at this time.

A large randomized clinical trial of the use of the AMPLATZER™ Cardiac Plug (Abbott Laboratories, Chicago, IL, USA) in comparison with OAC was halted following the approval of the WATCHMAN™ device (Boston Scientific, Natick, MA, USA) because it was believed it would be difficult to enroll patients in the trial. Most of the clinical data available for the AMPLATZER™ Cardiac Plug (Abbott Laboratories, Chicago, IL, USA) are derived from small single-center observational studies that were predominantly retrospective in design and sometimes involving a single operator. Most of the patients were treated with aspirin and clopidogrel during the postprocedural period, and the indication to enroll in the study was intolerance or contraindication to OAC with nonvalvular AF.^32–38^ The most frequent adverse events in this study were device embolization and pericardial effusion, which were very rare in the WATCHMAN™ study.^23^

Based on data from a major multicenter study (involving 22 centers and 1,047 patients), procedural success with the AMPLATZER™ Cardiac Plug (Abbott Laboratories, Chicago, IL, USA) was 97.3% (1,019 of 1,047 patients). Periprocedural SAEs numbered 52 (4.97%) during the follow-up period of 13 months (1,349 patient-years). There were nine (0.9%) strokes and nine (0.9%) transient ischemic attacks during the follow-up period. The rate of major bleeding was 1.5% (15 patients). Systemic thromboembolism occurred at an annual rate of 2.3% (31 of 1,349 patient-years), with a 59% risk reduction (the mean CHA_2_DS_2_-VASc score of implanted patients was 4.4 ± 1.6 with an annual risk for thromboembolism of 5.6% without the device; the 5.6% rate was reduced to 2.3% with device for a 59% risk reduction). This multicenter study of LAA occlusion with the AMPLATZER™ Cardiac Plug (Abbott Laboratories, Chicago, IL, USA) showed a favorable outcome for the prevention of systemic thromboembolism from AF and a high procedural success rate. Patients on aspirin monotherapy or no therapy had fewer bleeding events. Based on this study, the modification of antithrombotic therapy in the future with LAAO could result in fewer bleeding events^39^; however, additional research is still needed.

***Safety, efficacy, and comparison of the WATCHMAN™ device and AMPLATZER™ Cardiac Plug***. This was a single-center retrospective analysis of 165 patients (99 received the WATCHMAN™ device and 66 received the AMPLATZER™ Cardiac Plug) who underwent LAA closure. Notably, this was the first study to directly compare the WATCHMAN™ device (Boston Scientific, Natick, MA, USA) and the AMPLATZER™ Cardiac Plug (Abbott Laboratories, Chicago, IL, USA). Device selection and clinical indications were left to the operator’s discretion. Routine clinical and TEE follow-up was performed for a median period of 15 months. The ischemic events occurred at very low rates, and five patients died during the follow-up period. A higher incidence of severe [> 3 mm leak; 13 cases with WATCHMAN™ (18%) and four cases with the AMPLATZER™ Cardiac Plug (6.3%); p = 0.037] and moderate [> 1 mm leak; 17 cases with WATCHMAN™ (34%) and nine cases with the AMPLATZER™ Cardiac Plug (14%); p = 0.04] peridevice leak occurred with the use of the WATCHMAN™ device (Boston Scientific, Natick, MA, USA). This study proved the safety and efficacy of LAA closure devices; however, the clinical relevance of small peridevice flow needs further investigation.^40^

### AMPLATZER™ Amulet™

**Figure 3:**
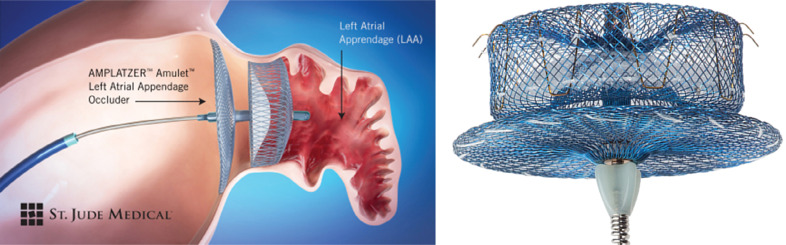
The AMPLATZER™ Amulet™ LAA occlusive device (Abbott Laboratories, Chicago, IL, USA). Image courtesy of Abbott Laboratories.

An investigational device exemption trial [AMPLATZER™ Amulet™ LAA Occluder Trial (Amulet IDE); NCT02879448] to evaluate the safety and effectiveness of the AMPLATZER™ Amulet™ LAAO device (Abbott Laboratories, Chicago, IL, USA) was initiated in 2016 and is currently in the recruitment stage. The device is designed to work by blocking the LAA at its opening and is believed to minimize the chances of blood clot migration into the bloodstream in patients with nonvalvular AF who cannot tolerate OAC. The AMPLATZER™ Amulet™ (Abbott Laboratories, Chicago, IL, USA) is a second-generation device with a longer lobe and waist than the AMPLATZER™ Cardiac Plug (Abbott Laboratories, Chicago, IL, USA). It is designed to allow for easier and more stable placement in a shorter procedure time **(Figure 3)**. The device is currently available in eight sizes to accommodate different anatomies.

The Amulet IDE trial is a randomized trial and is expected to enroll patients from up to 100 sites in the US and another 50 sites internationally. Patients enrolled in this trial will be randomly assigned to receive either a US FDA-approved LAA closure device in the control arm, or the AMPLATZER™ Amulet™ (Abbott Laboratories, Chicago, IL, USA) in the experimental arm. Data collected from across all trial sites will be used to support the potential FDA approval of the AMPLATZER™ Amulet™ LAAO device (Abbott Laboratories, Chicago, IL, USA).

The AMPLATZER™ Amulet™ (Abbott Laboratories, Chicago, IL, USA) has also demonstrated encouraging results in a prospective, single-center, side-by-side comparison study of consecutive patients undergoing LAAO with either the AMPLATZER™ Cardiac Plug (Abbott Laboratories, Chicago, IL, USA) or AMPLATZER™ Amulet™ (Abbott Laboratories, Chicago, IL, USA). A total of 59 patients (31 implanted with the AMPLATZER™ Cardiac Plug and 28 implanted with the AMPLATZER™ Amulet™) underwent LAAO during the study period, with the devices being successfully implanted in 58 patients. Follow-up TEE was subsequently performed in 50 patients (25 with the AMPLATZER™ Cardiac Plug and 25 with the AMPLATZER™ Amulet™; 86% of the total patient population). Based on this data, the AMPLATZER™ Amulet™ (Abbott Laboratories, Chicago, IL, USA) showed similar clinical and procedural outcomes with a significant reduction of any leak in comparison with its predecessor (48% leak with the AMPLATZER™ Cardiac Plug versus 8% with the AMPLATZER™ Amulet™).^41^ Further data with larger numbers of patients and long-term clinical outcomes are expected to be obtained with the completion of the Amulet IDE trial, which is employing the WATCHMAN™ device (Boston Scientific, Natick, MA, USA) in the control arm. Both the AMPLATZER™ Cardiac Plug (Abbott Laboratories, Chicago, IL, USA) and the AMPLATZER™ Amulet™ (Abbott Laboratories, Chicago, IL, USA) are available for use in Europe.

### The LARIAT^®^ procedure

**Figure 4:**
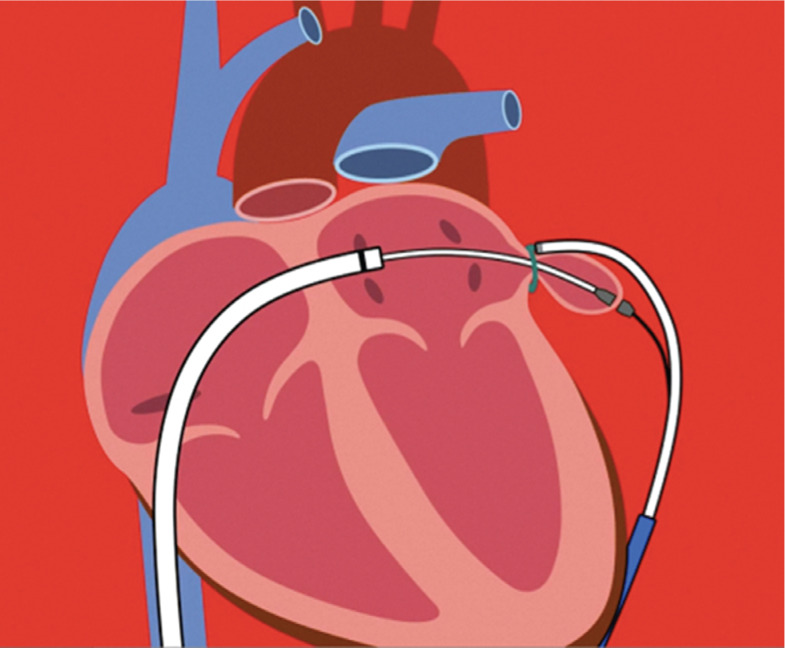
The LARIAT^®^ ligation device (SentreHEART, Redwood City, CA, USA). **Video 1** (available online) demonstrates the insertion process.

The LARIAT^®^ device (SentreHEART, Redwood City, CA, USA) enables ligation of the LAA by a transseptal and subxiphoid (epicardial) approach. In the procedure, surgical sutures are applied around the ostium of the LAA and approximate location of all walls, thus excluding the LAA via a transcatheter approach. The procedure involves transseptal and pericardial access. An endocardial magnetic-tipped guidewire is advanced to the apex of the LAA by balloon identification of the LAA ostium. Forming a rail by connecting the endocardial and epicardial guidewires, the suture can be advanced to capture the LAA **(Figure 4)**. The LARIAT^®^ procedure (SentreHEART, Redwood City, CA, USA) may be considered when the LAA is too large for the use of plugging devices, but the ostium has to be less than 40 mm for it to work. No anticoagulation is required, as there is no foreign body left behind on the endocardial surface of the left atrium. The LARIAT^®^ device (SentreHEART, Redwood City, CA, USA) was approved by the US FDA for soft tissue closure (approximation), but not specifically for LAA closure to prevent thromboembolism. LARIAT^®^ (SentreHEART, Redwood City, CA, USA) epicardial suture was also used for the termination of symptomatic persistent left atrial tachycardia arising from the LAA by electrical isolation of the LAA.^42^ LAA exclusion with the LARIAT^®^ procedure (SentreHEART, Redwood City, CA, USA) leads to persistent decrease in systolic blood pressure and an early decline in serum sodium levels, possibly because the LAA is a source of atrial natriuretic peptide, which plays an important role in homeostasis.^43^

The LARIAT^®^ procedure (SentreHEART, Redwood City, CA, USA) was successful in 96% of patients in an observational study involving 89 patients, with complete closure of the device confirmed in 98% of patients at one year postoperation. However, 25% of the initially screened patients were subsequently excluded due to LAA morphology, the presence of an LAA thrombus, and/or the onset of pericardial adhesions precluding pericardial access.^44^ The results of another multicenter study (a US transcatheter LAA ligation consortium) showed higher periprocedural complication rates. Major complications included significant pericardial effusion (10.4%)^22^ and major bleeding (9.7%).^45^ Device success (delivery of suture and residual leak of < 5 mm was achieved in 144 of 154 cases, or 94%) and overall procedure success was 86% (134 of 154 cases; periprocedural bleeding was the major reason for decreased overall procedure success).^45^

A multicenter observational study involving 344 patients (mean age: 70 years ± 10 years) considered the prevention of pericarditis during the LARIAT^®^ procedure (SentreHEART, Redwood City, CA, USA), sorting the patient cohort into 243 patients composing the colchicine group and 100 composing the standard group. The study suggested that periprocedural complications can be minimized significantly (the incidence of severe pericarditis was 4% in the colchicine group versus 16% in the control group; p < 0.0001). It also found that pericardial drain duration decreased by using colchicine prophylactically.^46^ Even using a micropuncture needle in comparison with using a conventional large-bore needle during epicardial access in the procedure was associated with decreased incidence of major complications, as seen in a separate multicenter observational study (0.9% versus 8.1%; p < 0.001).^47,48^

Furthermore, based on data from a third multicenter prospective observational study of note, in comparison with the WATCHMAN™ device (Boston Scientific, Natick, MA, USA), use of the LARIAT^®^ device (SentreHEART, Redwood City, CA, USA) is associated with a lower rate of residual leaks at one year postoperation, though, notably, there was no difference in the incidence rate of cerebrovascular accidents in the two device groups, despite the differences in residual leaks.^49^ LAA leaks from incomplete ligation following the LARIAT^®^ procedure (SentreHEART, Redwood City, CA, USA) are not uncommon and can be closed using the AMPLATZER™ septal occluder (Abbott Laboratories, Chicago, IL, USA) or via a repeat LARIAT^®^ procedure (SentreHEART, Redwood City, CA, USA).^50,51^

In the setting of a foreign body being present in the left atrium from endocardial occlusive device use, platelet aggregation leads to thrombus formation. This is more common with the use of endocardial occlusive devices than with the LARIAT^®^ (SentreHEART, Redwood City, CA, USA) endoepicardial approach. A nationwide survey of physicians who perform this procedure concluded that only 19 of 964 (2%) patients developed a left atrial thrombus, with antiplatelet and antithrombotic therapy initiated in these individuals following diagnosis.^52^ A possible reason for thrombus formation with the LARIAT^®^ procedure (SentreHEART, Redwood City, CA, USA) is focal endocardial damage and inflammation. Prompt initiation of anticoagulation can lead to thrombus resolution. This study strongly supported the performance of a follow-up TEE at 30 days to 90 days after the procedure.

In a prospective observational study involving 50 patients with AF and implanted cardiac devices who underwent the LARIAT^®^ procedure (SentreHEART, Redwood City, CA, USA), AF burden was found to decrease postoperatively. The presence of AF triggers in the LAA appears to be the strongest predictor of AF reduction (p < 0.0001).^53^ Notably, the US FDA issued a safety communication in July 2015 stating that complications associated with the LARIAT^®^ procedure (SentreHEART, Redwood City, CA, USA) include perforation of the heart and detachment of the LAA from the heart.^54^ Based on these data, only patients with absolute contraindications to anticoagulation and those who have anatomies unsuitable for endovascular closure should be considered for this procedure. More recently, the FDA also approved SentreHEART’s LAA Ligation Adjunctive to PVI for Persistent or Longstanding Persistent Atrial Fibrillation (aMAZE) clinical trial aimed at evaluating the LARIAT^®^ suture delivery system (SentreHEART, Redwood City, CA, USA) for closure of the LAA as an adjunct to ablation in patients with persistent or longstanding AF. The main objective of the aMAZE trial is to demonstrate that the use of the device in combination with pulmonary vein isolation ablation will reduce recurrent AF at a higher rate than pulmonary vein isolation ablation alone, which is the present standard of care.^55^

## Epicardial approach

### ATRICLIP^®^

The ATRICLIP^®^ device (AtriCure, Mason, OH, USA) is a clip made of two rigid, parallel titanium tubes with elastic nitinol springs covered with a knit-braided polyester sheath. During open cardiac surgery, this device is placed epicardially at the base of the LAA to prevent blood flow into it. The AtriCure Exclusion of the LAA in Patients Undergoing Concomitant Cardiac Surgery (EXCLUDE) trial^16^ was a nonrandomized, prospective multicenter trial that assessed the safety and efficacy of this device. The study was conducted in seven sites across the US with a total of 71 patients enrolled; the ATRICLIP^®^ device (AtriCure, Mason, OH, USA) was successfully placed in 70 of them, with follow-up occurring at three and 12 months postoperatively, respectively. This initial multicenter trial is notable because its results suggest that exclusion of the LAA can be achieved safely and without injury. During the short-term follow-up period, computed tomography angiography and/or TEE provided evidence of complete exclusion of the LAA in a significant number of study patients (98.4%).

Several other ongoing studies of note also incorporate the ATRICLIP^®^ device (AtriCure, Mason, OH, USA). The observational Stroke Feasibility Study (NCT01997905) is currently investigating the safety and anatomic efficacy of the ATRICLIP^®^ device (AtriCure, Mason, OH, USA) in patients with AF who have contraindications to OAC, while another trial—the AtriClip^®^ LAA Exclusion Concomitant to Structural Heart Procedures (ATLAS) study (NCT02701062)—is currently recruiting participants to evaluate the use of the ATRICLIP^®^ device (AtriCure, Mason, OH, USA) in patients undergoing a valve or coronary artery bypass graft procedure. Additionally, another study recruiting patients is the Combined Endoscopic Epicardial and Percutaneous Endocardial Ablation Versus Repeated Catheter Ablation in Persistent and Longstanding Persistent AF (CEASE-AF) trial (NCT02695277), wherein the ATRICLIP^®^ device (AtriCure, Mason, OH, USA) will be used in the experimental arm of the study to evaluate the efficacy and safety of two interventional approaches in preventing the recurrence of AF in symptomatic, drug-refractory patients with persistent or longstanding persistent AF.

## Other left atrial appendage occlusion devices

**Figure 5:**
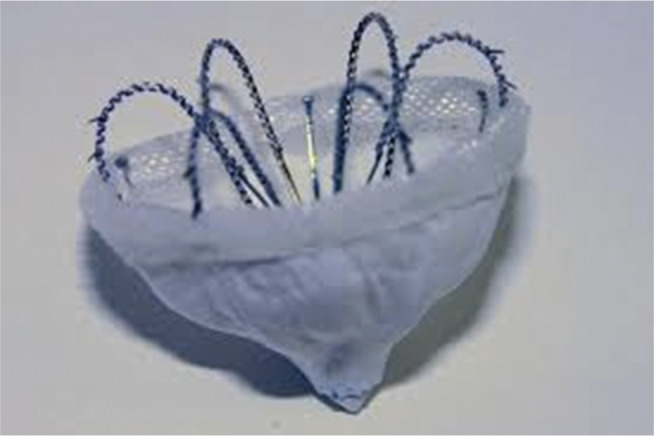
The Coherex WaveCrest™ LAA occlusion system (Coherex Medical, Salt Lake City, UT, USA).

There are several other LAAO devices currently in different stages of development and/or clinical trials. These include:

1. The Coherex WaveCrest™ LAA occlusion system (Coherex Medical, Salt Lake City, UT, USA), a polytetrafluoroethylene cap/occluder with distal anchors **(Figure 5)**. During device implantation, the proximal expanded polytetrafluoroethylene cap/occluder is positioned, the distal anchors are deployed, and foam is incorporated into the edges of the occluder to enhance the LAA sealing. Though this device is available for use in Europe, it is still in the testing phases in the US. One recently completed trial (NCT02239887; results pending) sought to establish the safety and efficacy of the device for LAA closure in patients being treated for nonvalvular AF who are at increased risk for embolic stroke and who have an ongoing indication for oral anticoagulation.
2. The LAmbre™ LAA occluder (Lifetech Scientific Corp., Shenzhen, China) is a self-expanding nitinol device consisting of a distal, hook-embedded umbrella and a proximal covering disk. A sewn-in polyethylene terephthalate fabric covers both the umbrella and the proximal disk, and a short central waist connects the cover and the umbrella. This first LAA closure system from China has received CE mark approval for use in Europe, but has not yet been approved for use in the US. Several initial studies have indicated its possible feasibility, safety, and efficacy in canines and in humans, respectively.^56–58^
3. The Occlutech LAA Occluder (Occlutech International AB, Helsingborg, Sweden) is a self-expanding, cone-shaped device anchored with closed loops at the distal device margin. It is approved in Europe. Several preclinical assessments have suggested the device’s good performance in canine and swine models, respectively^59,60^; a nonrandomized study investigating the safety and efficacy of the device for percutaneous LAA closure in adult patients with AF is also ongoing.^61^
4. The Ultrasept LAA Occluder (Cardia Inc., Eagan, MN, USA) is made from a nitinol frame with a distal cylindrical anchor that is deployed within the appendage and secured therein using platinum/iridium collars and polyvinyl alcohol foam, which orientates onto and covers the ostium. The device’s long and flexible waist allows for increased positional versatility and the stranded design of its frame increases fatigue resistance and enables fine-tuning of the tension applied to the sail and anchor to occur. The sail is designed to minimize blood flow disturbance within the LAA. The Ultrasept LAA Occluder (Cardia Inc., Eagan, MN, USA) is available in five sizes. Currently, no human data appear to be available, though the Canadian LAA Closure Study (CLASS) is currently recruiting participants.^62,63^
5. A purely epicardial system (Sierra Ligation System; Aegis Medical, Los Angeles, CA, USA) remains investigational at this time, with a feasibility trial currently recruiting participants.^64^ In earlier studies, a percutaneous electrogram-based approach to capture and ligate the LAA via the use of a hollow suture preloaded with a mechanical support wire used to approximate under fluoroscopic guidance was successfully completed in dogs and achieved complete closure and fibrosis, with a remnant atretic LAA noted in all animals.^65,66^ Additionally, a multicenter study on the efficacy and safety of LAA closure using an epicardial suture snaring device was expected to begin last year.^67^

## Conclusions and future directions

AF has been increasing in prevalence globally. OAC and NOACs are currently the first line of treatment for the prevention of thromboembolism in AF; however, in spite of their proven efficacy, there are several limitations associated with their use, including the risk of bleeding.

**Figure 6:**
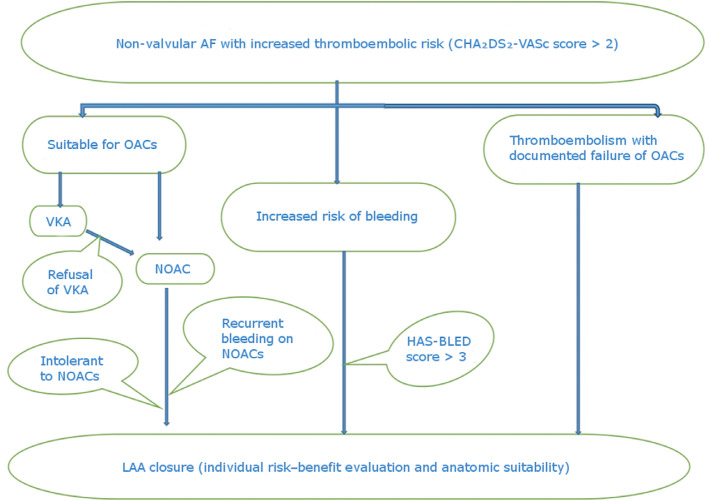
An algorithm of stroke prevention in AF. AF: atrial fibrillation; LAA: left atrial appendage; NOAC: novel oral anticoagulant; OAC: oral anticoagulant; VKA: vitamin K antagonist.

The LAA is considered to be the major source of thrombus (> 90%) in nonvalvular AF and is the prime target for transcatheter interventional therapy with device occlusion or ligation in patients who are intolerant of OAC therapy **(Figure 6)**. LAA occlusion by interventional techniques has been proven to be noninferior to oral VKA use in reducing thromboembolic events.^68^ The use of NOAC and dual antiplatelet therapies for a short period of time (45 days) was also proven to be safe and effective following interventional LAA closure.^69^

Based on recent European Society of Cardiology guidelines (2016), LAA occlusion can be considered (class IIb, LOE A) to prevent stroke in patients with AF and contraindications to long-term anticoagulant therapy (eg, patients who have experienced potentially fatal bleeding without a reversible cause).^70^

Based on an observational study, other advantages of LAA exclusion in patients with AF and hypertension include a significant reduction in blood pressure and a requirement of antihypertensive drugs at three months and 12 months after the procedure. There is a need for further research to evaluate this therapy in multidrug-resistant hypertension patients.^71^

The benefit of LAA closure in reducing the incidence of stroke in patients with nonvalvular AF has been evolving gradually, but no device has thus far been studied adequately in a randomized controlled fashion in a high-risk AF population that is eligible for treatment with anticoagulation. The Left Atrial Appendage Closure vs. Novel Anticoagulation Agents in Atrial Fibrillation (PRAGUE-17) trial^72^ is an ongoing multicenter, randomized controlled study comparing LAA occlusion with NOAC treatment in AF patients at high risk for cardioembolic events.

Newer techniques need to be developed to increase safety and decrease procedure times. Further continued prospective studies are still necessary to evaluate the long-term efficacy and to determine a specific anticoagulation or antiplatelet regimen and duration. Based on these encouraging results from LAA closure devices in nonvalvular AF, there is a need for prospective randomized research to assess the efficacy of these devices in patients with valvular AF who are receiving treatment with percutaneous valve replacement. The “one-stop shop” approach of combining percutaneous valve replacement or mitral clipping and LAAO in patients with valvular abnormalities with AF has been shown to be feasible in case reports,^73,74^ but needs to be considered in a prospective study to confirm and prove its safety and efficacy.

In conclusion, we are confident that this new field of percutaneous LAAC will continue to evolve and potentially act as a game-changer in the treatment of AF.

## Figures and Tables

**Table 1:** Randomized Clinical Trials Comparing WATCHMAN™ with Warfarin in Patients with Nonvalvular AF and Their Efficacy Outcomes

Study	Number of Patients	Patient Characteristics	Control	Primary Endpoint	Duration of Follow-up	Treatment Effect	NI Achieved?
PROTECT AF^12^	707	Warfarin-eligible; CHADS_2_ score ≥ 1	Warfarin	Cardiovascular death, any stroke, or systemic embolism	1,065 patient-years	RR: 0.62 (0.35–1.25)	Yes
					1,588 patient-years	RR: 0.71 (0.44–1.30	Yes
					2,621 patient-years	RR: 0.60 (0.41–1.05)	Yes
						NI margin (upper bound of 95% Crl): RR < 2.0	
PREVAIL^23,24^	407	Warfarin-eligible; CHADS_2_ score ≥ 2, or CHADS_2_ score = 1 plus an additional RF ablation	Warfarin	Cardiovascular death, any stroke, or systemic embolism	11.8 months ± 5.8 months	RR: 1.07 (0.57–1.89); NI margin (upper bound of 95% CrI): RR < 1.75 Difference: 0.0053; NI margin (upper bound of 95% CrI); rate difference < 0.0275	No
				Any stroke or systemic embolism > 7 days after the procedure			Yes

AF: atrial fibrillation; NI: noninferiority; RR: rate ratio; CrI: credible interval; RF: radiofrequency.
